# An Integrated Approach to Skeletal Muscle Health in Aging

**DOI:** 10.3390/nu15081802

**Published:** 2023-04-07

**Authors:** Deborah Agostini, Marco Gervasi, Fabio Ferrini, Alessia Bartolacci, Alessandro Stranieri, Giovanni Piccoli, Elena Barbieri, Piero Sestili, Antonino Patti, Vilberto Stocchi, Sabrina Donati Zeppa

**Affiliations:** 1 Department of Biomolecular Sciences, University of Urbino Carlo Bo, 61029 Urbino, Italy; 2 Sport and Exercise Sciences Research Unit, Department of Psychology, Educational Science and Human Movement, University of Palermo, 90128 Palermo, Italy; 3 Department of Human Science for Promotion of Quality of Life, Università Telematica San Raffaele, 00166 Rome, Italy

**Keywords:** musculoskeletal health, aging, vitamin D, physical exercise, supplements, gut microbiota

## Abstract

A decline in muscle mass and function represents one of the most problematic changes associated with aging, and has dramatic effects on autonomy and quality of life. Several factors contribute to the inexorable process of sarcopenia, such as mitochondrial and autophagy dysfunction, and the lack of regeneration capacity of satellite cells. The physiologic decline in muscle mass and in motoneuron functionality associated with aging is exacerbated by the sedentary lifestyle that accompanies elderly people. Regular physical activity is beneficial to most people, but the elderly need well-designed and carefully administered training programs that improve muscle mass and, consequently, both functional ability and quality of life. Aging also causes alteration in the gut microbiota composition associated with sarcopenia, and some advances in research have elucidated that interventions via the gut microbiota–muscle axis have the potential to ameliorate the sarcopenic phenotype. Several mechanisms are involved in vitamin D muscle atrophy protection, as demonstrated by the decreased muscular function related to vitamin D deficiency. Malnutrition, chronic inflammation, vitamin deficiencies, and an imbalance in the muscle–gut axis are just a few of the factors that can lead to sarcopenia. Supplementing the diet with antioxidants, polyunsaturated fatty acids, vitamins, probiotics, prebiotics, proteins, kefir, and short-chain fatty acids could be potential nutritional therapies against sarcopenia. Finally, a personalized integrated strategy to counteract sarcopenia and maintain the health of skeletal muscles is suggested in this review.

## 1. Introduction

A decline in muscle mass and function represents one of the most problematic changes that occur with aging, leading to dramatic effects on the subject’s capacity for autonomy and their quality of life [1]. Age-related muscle loss has been studied since the early 1970s [2]; recent prospective studies have obtained more precise estimates of annual skeletal muscle loss, estimated to be between 0.65% and 1.39% for elderly men and between 0.61% and 0.80% for elderly women, using dual-energy x-ray absorptiometry (DXA) [3]. Age-related decline in muscle mass, strength, and function was first named “sarcopenia” in 1989 by Irvin Rosenberg, and has become a major global health concern, especially as a result of the exponential increase in the elderly population worldwide [4].

The operational definition of sarcopenia given by the European Working Group on Sarcopenia in Older People (EWGSOP) in 2010 [5] has evolved from that established in the past, and proposes an algorithm for the diagnosis of sarcopenia based not only on the presence of low muscle mass, but also low muscle function (strength and muscle performance); in the 2018 update (EWGSOP2), low muscle strength was indicated as the main parameter for identifying pre-sarcopenic conditions, sarcopenia, and severe sarcopenia [6]. In 2016, sarcopenia was recognized as a muscle condition/disease and received its ICD-10-CM (International Classification of Diseases-10-Clinical Modification) code (M62.84), leading to a major breakthrough in the recognition of sarcopenia as an illness [7].

Skeletal muscle can adapt to numerous stresses, including pathological ones, through changes in metabolism, fiber composition, or size [8]. Such adaptations, or a lack of them, especially during aging, can lead to atrophy, or a significant loss of muscle mass, with consequent problems for the health of older people. An attempt has been made to explain the development of sarcopenia by proposing the involvement of different biological mechanisms [9].

Mitochondrial dysfunction plays an important role in the muscle aging process, both in rodents and in humans [10,11]. The mitochondrial content in the muscle is the result of alternation in the synthesis (biogenesis) and degradation (mitophagy) pathways activated by different stimuli [12]. These organelles participate in cellular signaling pathways through the production of reactive oxygen species (ROS) [13], as well as being mediators of apoptosis [14,15].

Dysfunctional mitochondria result in three main age-related changes: a reduced ability to produce ATP, and therefore, less energy for cells [16]; increased susceptibility to apoptosis [17,18]; and increased generation of ROS [19,20]. 

Oxidative stress is one of the causes of sarcopenia [21], and aging increases the production of ROS [22] and lowers the levels of antioxidant enzymes in muscle and neuronal cells [23] by damaging cellular mitochondria [24]. Regardless of the source of production, excess ROS contributes to the production of damaged and dysfunctional mitochondria, leading to the release of mitochondrial proteins into the cytosol [25]; this may initiate the signaling cascade, leading to the apoptosis of skeletal muscle cells [26,27,28,29].

It has also been seen that good functioning of autophagy is necessary to maintain the quality and quantity of muscle [30]; in fact, the genetic inhibition of some regulators, such as the autophagy-related proteins ATG7 or ATG5, causes a loss of muscle mass and the accumulation of poor-quality mitochondria [30,31]. Some studies have shown that the activation of the ubiquitin–proteasome system (UPS) leads to an increase in protein degradation, playing a role in sarcopenia [32]. The muscle wasting-related proteins MuRF-1 and Atrogin-1 are both UPS E3 ligases, and are activated during the muscle wasting process [33,34].

Satellite cells (or muscle stem cells) are of particular importance in the repair and maintenance of muscle fibers. Satellite cells are able to carry out muscle repair or hypertrophy, or return to being inactive [35]. In adult muscle, the number of satellite cells (SCs) always remains rather constant [36], creating a safety reservoir to draw on if needed. SCs, in addition to being powerful myogenic progenitors, are capable of self-renewal to keep their population constant, or even increase it when necessary [37]. Another essential element for muscle regeneration is the presence of the growth factor insulin-like growth factor 1 (IGF-1), which is associated with all phases of regeneration and which, acting on multiple pathways, promotes the proliferation, differentiation, and survival of SCs [38]. In very old rodents, it has been observed that, also due to an increase in ROS, some satellite cells develop a state of irreversible pre-senescence alongside a loss of normal quiescence (normally reversible). This condition definitively affects their intrinsic ability to self-regenerate and, in the event of injury, to activate themselves to give rise to repair [39]. The lack of regeneration capacity of the sarcopenic muscle, in addition to being a hallmark of aging, is one of the main causes of loss of independence in elderly subjects [40,41,42].

The strong sedentary lifestyle that accompanies aging predisposes the elderly to an accumulation of fat not only in the normal deposit sites, but also in the tissues of the organs (ectopic fat) and within skeletal muscle (intermuscular adipose tissue (IMAT)) [43]. During old age, IMAT leads to inflammatory and metabolic problems [44], and also to mitochondrial dysfunction, leading to a reduced fatty acid oxidation capacity [45] and an increase in oxidative stress and insulin resistance (IR) [46], with a consequent decrease in the quantity and quality of muscle mass [47]. Alongside the decline in muscle mass, there is also a decline in motoneuron functionality and nerve transmission. In this complex scenario, there are various pharmacological and non-pharmacological strategies that are useful for preventing and improving this disease. 

In this review, we analyze several non-pharmacological interventions, such as exercise, microbiota modulation, nutritional modifications, and supplementation with antioxidants, polyunsaturated fatty acids (PUFAs), vitamins, probiotics, prebiotics, proteins, kefir, and short-chain fatty acids, in order to propose an individualized and integrated approach that aims to preserve and ameliorate musculoskeletal health.

## 2. Physical Exercise and Muscle Health

Low levels of physical activity are among the main risk factors for sarcopenia, along with the muscle fiber decline that begins in midlife [48,49]. This is important from a clinical standpoint, as it leads to reduced strength and exercise capacity, both of which are required to undertake normal daily living activities. This loss of skeletal muscle mass, which accelerates from the age of 60 to 80 years, is predictive of all-cause mortality, regardless of age, BMI, lifestyle, physical performance, health status, or body composition [50].

According to the literature, age-related muscle mass loss is thought to be caused by a complex set of factors that determine the degradation of motoneurons, leading to the complete atrophy of motor units. Specifically, the degenerative process of motor end plates causes slower and decreased nerve transmission, followed by denervation that, combined with muscle cell atrophy, causes permanent harm to striated muscle tissues [51].

On the other hand, exercise appears to have therapeutic benefits that counteract these processes. A study on mammals showed that four months of endurance exercise dramatically slow down the loss of nuclear pore complex proteins in elderly animals, while preserving the integrity of the neuromuscular junction and delaying the loss of motoneurons specifically [52]. In particular, the key neurotrophic factors, neurotrophin-4 (NT-4) and the brain-derived neurotrophic factor (BDNF), which both play a role in modifying and sustaining the neuromuscular synapse, are upregulated in skeletal muscle after exercise. The increased production of BDNF, and its regulation via regular exercise and/or supplementation, are dependent upon redox-sensitive pathways and oxidative stress status [53,54]. 

Such benefits at the muscle and motoneuron levels imply that exercise is related to both cell health and neuromuscular junction maintenance [55,56,57].

Furthermore, with age, higher-conduction-velocity fibers (type 2 fast-twitch fibers) may be innervated by smaller motoneurons as a result of the reinnervation of fibers, leading to an increase in muscle fatigability in the elderly [58,59]. However, in this case, physical activity can also play a crucial role, minimizing the problem by stimulating the innervation of new fibers. In this regard, findings by Mosole et al. [60,61] showed that long-term, intense exercise promotes the reinnervation of muscle fibers, with positive effects on muscle structure and function, ultimately leading to a delay in mobility decline.

Another key indicator of the loss of muscle mass in aging seems to be a lack of monoaminergic input to the motoneurons [62]. The absence or a decrease in this input can reduce the persistent inward currents (PICs) of the voltage-gated persistent Na+ and Ca2+ channels located on motoneuron dendrites and soma, increasing the firing threshold for turning on motoneurons, and thus, causing muscle weakness in the elderly [63,64]. However, evidence from Vila-Cha et al. [65], showed that motoneuron firing rates increase after six weeks of resistance training and decrease after endurance training.

Another factor, perhaps the most crucial one, responsible for the inexorable decrease in muscle mass with aging, is the presence of significantly fewer adult skeletal muscle stem cells (SMSCs) in older subjects. However, it is unclear whether this decline in SMSCs is due to the intrinsic mechanisms of aging within cells or extrinsic environmental changes; hormonal immunologic and metabolic problems are frequent environmental variables associated with aging, and are thought to have a significant impact on stem cell function [66,67]. The alteration in stem cell activity with aging is thought to be caused by changes in these microenvironmental variables in response to aging [68]. Indeed, hormonal abnormalities brought on by endocrine gland aging have been linked to altered stem cell activity and/or differentiation [69]. Hormonal changes, particularly changes in estrogen levels, seem to be the main changes that occur as humans age. Additionally, estrogen deficiency causes SMCs to differentiate preferentially into adipocytes rather than osteoblasts [66,70,71]. SMSC differentiation abnormalities can also result from an increase in circulating amounts of pro-inflammatory mediators associated with aging, such as interleukin 6 (IL-6) and tumor necrosis factor (TNF-α) [72,73]. Other elements, such as growth hormone (GH), insulin-like growth factor (IGF-1), and androgens that assist in regulating skeletal muscle growth and development, seem to decline with age [69].

The role that physical activity can play is precisely that of maintaining this microenvironment as efficiently as possible by stimulating the production of anti-inflammatory, hormonal, and neuroendocrine factors that slow down the muscle aging process [74]. However, although physical activity recommendations (IPAR) may slightly slow down the processes related to sarcopenia, a recent meta-analysis [75] showed that low-intensity resistance training (≤50% 1RM) is insufficient to induce strength gains, and the authors recommend a high-intensity resistance training program (i.e., 80% 1RM) and a multimodal exercise approach. Indeed, while regular physical activity is beneficial to most people, older people need to exercise in a way that is tailored to their unique needs and abilities. Several different types of exercise have been shown to reduce the risk of developing sarcopenia and improve strength [76,77]. When selecting exercises for the elderly, it is vital to examine the cross-relationship between resistance and endurance training, as well as the proper kind of training and its impact on muscle mass. Resistance training increases muscle mass [78,79], while endurance training can sometimes decrease that mass [80]. The key to success for both Masters athletes [81] and previously sedentary elderly individuals is adopting a well-designed and carefully administered training program [49,82] that improves muscle mass and, consequently, both functional ability and quality of life [83]. Important gaps in research remain, including a lack of studies on the benefits of group interventions and the possible benefits of professionals delivering interventions [77]. To this end, training protocols developed and structured by experts who are able to customize the intensity and optimal amount of resistance and endurance training are recommended. Such protocols should include flexibility and proprioceptive exercises [77].

## 3. Microbiota and Muscle Health

The human intestine is colonized by numerous microorganisms, which are considered critical for the proper performance of many physiological functions, such as maintaining intestinal mucosal integrity, regulating host immunity, and maintaining metabolic health [84]. The gut microbiota is usually shaped in early childhood, and the microbial composition and metabolism are constantly influenced by several factors, such as diet, drugs, physical exercise, and social environment [85].

In healthy individuals, two major bacterial phyla, *Firmicutes* and *Bacteroidetes*, account for approximately 99% of the microorganism species in the colon. However, the composition of the gut microbiota is found to be altered during aging or disease [85].

The composition and properties of the microbiome may depend on their location, as microbial populations on the mucosal surface and in the gastrointestinal lumen interact with the host immune system, and are influenced by the metabolic effects of food [86]. The gut microbiome plays a critical role in the formation and maturation of the immune system, through the prevention of pathogen colonization, the stimulation of immunoglobulin A production, the upregulation of anti-inflammatory cytokines, and the regulation of T-lymphocyte cells. For example, *Faecalibacterium prausnitzii* and *Bifidobacterium infantis* can elicit the production of the anti-inflammatory cytokine interleukin-10 (IL-10) and regulate T cell activation against the pathogen-stimulated nuclear factor kappa-light-chain-enhancer of the activated B cell (NF-κB) inflammatory pathway [87]. Other species can also induce a reduction in levels of inflammation through the expression of interleukin-17 (IL-17), aiding the host’s immune system in protecting them against harmful pathogens. Furthermore, the gut microbiome is crucial in the de novo synthesis of essential vitamins, such as vitamin B12, folic acid, vitamin K, nicotinic acid, pyridoxine, and others, as well as bile acids. Impaired co-metabolism of bile acids and vitamins is associated with the development of metabolic diseases, such as obesity and type 2 diabetes [88]. A list of all the functional capacities of the human gut microbiome quantified 9,879,896 genes across 1267 stool samples from individuals of various geographical origins (Europe, USA, and China) and diverse health status (healthy, obese, diabetic, with inflammatory bowel disease, etc.), suggesting that the composition of the gut microbiota is influenced by multiple factors, such as host genetics, diet, health status, aging, and antibiotic administration [89]. In response to these stimuli, the microbial community adapts, changing its bacterial composition and function throughout the life of the human organism.

Some scientific evidence has shown that the gut microbiota profile correlates with changes in muscle mass [90]. The concept of the ‘gut–muscle axis’ has been raised to study this relationship [91]. Baeckhed and colleagues were the first to hypothesize the existence of the gut microbiota–muscle axis, demonstrating that germ-free (GF) mice are protected from diet-induced obesity by two mechanisms that lead to increased fatty acid catabolism in muscle [92].

This increase in whole body weight can be attributed, at least in part, to the increased weight of the appendix in these GF mice. GF mice show atrophy of skeletal muscle with a decrease in muscle growth factors, such as IGF-1, amino acids alanine and glycine, and a decrease in mitochondrial function [93].

Muscle physiology can be influenced by metabolites produced by the gut microbiota, such as linoleic acids, acetate, bile acids, and microbial molecules able to modulate the production of pro-inflammatory cytokines [94].

An increase in energy expenditure in human skeletal muscle and a decrease in muscle fat deposition are promoted by bile acids through the activation of the nuclear farnesoid X receptor [95], and by promoting intracellular thyroid hormone activation via TGR5, a G protein-coupled receptor [96].

Antibiotic-treated mice showed increased fatigue and decreased endurance exercise capacity [97]. 

Scheiman et al. reported that the inoculation of *Veillonella atypica* from marathon runners’ stool samples into mice significantly increased exhaustive treadmill run time. *Veillonella* species metabolize lactate into acetate and propionate via the methylmalonyl-CoA pathway. The genus *Veillonella* and its metabolic pathway, which converts lactate into propionate, is enriched in athletes after exercise. The intrarectal instillation of propionate has been sufficient to reproduce the increased treadmill run time performance observed with *V. atypica* gavage, thereby identifying a natural, microbiome-encoded enzymatic process that enhances athletic performance [98].

Lahiri et al. demonstrated that the gut microbiota can influence factors involved in muscle functionality, such as IGF-1 and mitochondria, leading them to hypothesize a central role of microbiota in influencing and maintaining muscle functionality [93].

The muscle strength of mice colonized with the microbiota of high-functioning older adults is increased. This suggests that the family *Prevotellaceae*, the genera *Barnesiella* and *Prevotella,* and the species *Barnesiella intestinihominis* are involved in mechanisms related to the maintenance of muscle strength in older adults [99]. 

Toll-like receptors (TLRs)/the NF-κB signaling pathway could hypothetically be involved in the gut microbiota–muscle axis. Muscle-specific activation of the transcription factor NF-κB leads to muscle wasting [100]. NF-κB is an important downstream target of TLRs that recognize various pathogen-associated molecular patterns (PAMPs). In particular, muscle cells respond to the TLR-2, -4, and -5 ligands [101]. Interestingly, TLR4 mediates muscle atrophy induced by LPS injection [102]. The involvement of TLR2 and TLR5 in muscle atrophy should be investigated. 

Finally, it is conceivable that microbiota-related inflammation occurs in several cachectic diseases and malnutrition [103,104]. These pathologies might be associated with altered gut barrier function [105], which could, in turn, lead to an increased translocation of PAMPs prone to inducing inflammation and muscle atrophy. A similar process, namely increased LPS translocation, has been proposed as a driver of inflammation associated with obesity and type 2 diabetes [106].

Aging causes alterations in the gut’s bacterial composition, such as an increase in proteolytic microorganisms or a decrease in saccharolytic bacteria, which are associated with sarcopenia [107].

Recent advances elucidated that interventions via the axis have the potential to reverse the sarcopenic phenotype [108].

The gut microbial diversity and muscle mass of male rats at different ages (8, 18, and 24 months) showed that the age-related alteration of gut microbiota led to a loss of muscle mass and function in aged mice [109].

Treatment with *Lactobacillus reuteri* led to the restoration of lost muscle mass induced by aging or by cancer through the phenomenon of cachexia, suggesting that alteration of the gut microbiota could help compensate for or counteract sarcopenia [110].

Furthermore, 12 weeks of *L. casei* LC122 or *B. longum* supplementation improved muscle function, endurance capacity, and lipid metabolism and reduced inflammation and oxidative stress in aged mice [111]. These results are associated with improved learning and memory performance and the upregulation of neurodegenerative and neurotrophic factor expression in the hippocampus, emphasizing the central role of gut–brain communication.

There are numerous mechanisms through which the gut microbiota and brain can exchange information via continuous, bidirectional communication; the detection and transduction of information from the gut to the nervous system involve neural, endocrine, and inflammatory mechanisms [112].

Malnutrition in older adults and in patients with chronic diseases is a major trigger for alteration of the gut microbiota, and regulation of the diet plays an important role in remodeling the microbiome and microbiome-derived micronutrients and metabolites, which, in turn, can cross the gut barrier and reach and affect muscle [108]. Lipopolysaccharide (LPS) and trimethylamine N-oxide, for example, induce proinflammatory status, whereas short-chain fatty acids (SCFAs) and bile acids regulate host metabolism [113]. 

In addition, an alteration in the composition of the gut microbiota was observed in people older than 65 years compared with that more commonly found in younger adults [114,115].

*Bifidobacterium* and *Lactobacillus* lead to increased microbial diversity that can promote more efficient nutrient uptake and amino acid synthesis, while excess nutrient uptake and storage and an increased *Firmicutes/Bacteroidetes* ratio is associated with low microbial diversity [116,117].

An increased abundance of *Firmicutes* relative to *Bacteroides* concentration is found in the microbiomes of obese and insulin-resistant humans and animals [118,119].

Interestingly, during aging, *Bifidobacterium* levels decrease, which is related to increased circulating levels of LPS. In obese and diabetic individuals, LPS endotoxin levels are significantly elevated, resulting in dysbiosis of the gut microbiota, increased intestinal permeability, and skeletal muscle insulin resistance [120,121,122,123,124].

The promotion of musculoskeletal resistance to insulin is due to the presence of LPS, a marker of endotoxemia, which, through activation of the Toll like receptor-4 (TLR-4) pathways, leads to the final expression of proinflammatory cytokines such as TNF-α, interleukin-1 (IL-1), interleukin-2 (IL-2), and IL-6 [125,126]. 

This highlights how an age-related decrease in the gut microbiota (e.g., *Bifidobacterium*) potentially influences the development of sarcopenic obesity through decreased glucose tolerance in skeletal muscle [117,126,127,128]. 

Thus, the production of inflammatory cytokines led to the suppression of protein synthesis through an imbalance between muscle protein synthesis and degradation (MPS: MPB), resulting in reduced muscle mass and physical function [129,130,131,132]. In addition, serum LPS and TLR4 levels in older adults are linked to lower insulin sensitivity than in younger adults, showing that age-related LPS levels may increase the incidence of insulin resistance during aging [133,134].

Therefore, reduced microbial diversity and function can directly affect skeletal muscle [132,135,136]. However, the important question is whether the microbiome is responsible for the consequences of aging or whether the alteration in microbial diversity is caused by aging [137,138].

## 4. Nutrition and Supplements

A well-balanced diet is important for musculoskeletal health because it provides energy, macronutrients, vitamins, and minerals. However, older people consume less energy and protein than younger people, despite having higher nutritional needs [139].

An increased intake of micronutrients, such as antioxidant nutrients and other plant bioactive compounds, is provided by the Mediterranean dietary pattern, which is characterized by high consumption of fruit, vegetables, and plant-based foods (legumes, nuts, and seeds), the use of olive oil, and lower consumption of meat and dairy foods. Compliance with this pattern may also be linked to a number of health benefits, including improved gut microbiota status, that are closely related to preserved muscle mass and improved physical performance in aging populations [140].

The role of the diet in sarcopenia-related inflammation in Crohn’s disease (CD) patients is of great importance due to the significance of inflammation in the etiology/pathophysiology of sarcopenia [141]. 

Pro-inflammatory processes, linked to the gut microbiota, are caused by consuming ultra-processed foods (UPFs), defined according to the recent NOVA classification system [142]. 

Highly processed foods include industrially processed pastries, meat and dairy products, savory snack foods, and sugar-sweetened beverages, and are typically energy-dense and low in nutrient content; however, unfortunately, their hyper-palatable attributes promote their overconsumption. These foods frequently represent bad eating habits and have negative health impacts, including all-cause mortality, cardiovascular disease, metabolic syndrome, cancer, and other pathological conditions [143]. These illnesses are also associated with oxidative stress and inflammation, which may alter the composition, variety, and richness of the gut microbiota [144]. Due to the fact that short-term modification in dietary patterns can impact gut microbiota variety and composition, nutrition plays a fundamental role in bacterial homeostasis.

Specific dietary components that have anti-inflammatory actions, such as n-3 fatty acids, have been demonstrated to possess protective effects [145].

On the other hand, poor eating habits can lead to obesity through massive modification of the gut microbiota. Consequently, alteration of the gut–muscle axis makes obesity an important potential cause of sarcopenia [146].

With the increase in evidence directly linking malnutrition, chronic inflammation, and an imbalance in the muscle–gut axis, several nutritional supplements have emerged as potential integrated therapies against sarcopenia (Table 1). For example, low vitamin D levels are predictive of an increased risk of sarcopenia in the elderly [147], and its supplementation can greatly help. 

We describe the preventive and therapeutic properties of antioxidants, PUFAs, vitamins, probiotics, prebiotics, proteins, kefir, and SCFAs, with the aim of suggesting an integrated approach to preventing and supporting musculoskeletal health in aging.

### 4.1. Vitamin D

Vitamin D (vit D) levels are negatively correlated with the risk of a number of diseases, including cancer, cardiovascular disorders, obesity, and sarcopenia. Epidemiological data supporting the link between vit D and an elevated risk of sarcopenia in older persons were evaluated by Remelli et al. [147]. Approximately 80% of vit D can be produced via irradiation with ultraviolet light B (UVB) starting from 7-dehydrocholesterol, which is converted into pre-vitamin D3, and then, to vit D3 (cholecalciferol.) The remainder is introduced as D3 or ergocalciferol (D2). These values may vary based on factors such as ethnicity, length of solar exposure, and season [175]. In the liver cytochrome P450, family 2 subfamily R member 1 (CYP2R1) or family 27 subfamily A member 1 (CYP27A1) hydroxylate vit D at the C25 site, producing 25-hydroxyvitamin D (25(OH)D). In the kidney, CYP27B1 hydroxylates 25(OH)D at the C1 site to form 1,25-dihydroxyvitamin D (1,25(OH)2D). The 1,25(OH)2D then binds the vit D receptor (VDR), a nuclear ligand-dependent transcription factor that regulates the expression of VDR target genes, causing several physiological responses. CYP27B1 is not only expressed in the kidney, but it is also widely expressed outside of it [176], increasing local site-specific concentrations of 1,25(OH)2D, which are then able to interact with endogenous VDR in the same tissue [177]. The primary mechanism for the 1,25(OH)2D -VDR-mediated modulation of gene transcription in multiple extra-renal organs appears to be this intracrine synthesis of 1,25(OH)2D. 25(OH)D and 1,25(OH)2D are metabolized, inactivated, and partially eliminated in urine or bile.

Vit D deficiency, decreased muscular function, and a higher incidence of sarcopenia have all been linked in numerous human studies [178]. Around one billion people are thought to be vit D-deficient worldwide [179].

Age raises the risk of vit D insufficiency and abnormal vit D function due to poor diet, reduced cutaneous vit D synthesis impairment, or changed expression of vit D metabolic enzymes and reduced VDR expression in skeletal muscle [180].

Vit D is best known for its role in regulating calcium homeostasis and bone health, but several mechanisms are involved in vit D muscle atrophy protection. The ubiquitin proteasome pathway is upregulated in atrophy, and increased expression of atrogin-1 and MuRF1, two E3 ubiquitin ligases, is considered a marker of cachexia [181]. In differentiated human myotubes and C2C12 models of stress, treatment with vit D suppresses atrogin-1 and muscle RING-finger protein-1 (MuRF1) expression [182,183]. A loss of AMPK with aging is harmful because it makes cells less able to respond to conditions of metabolic dysregulation, which can lead to an increase in oxidative stress. 1,25(OH)2D therapy appears to boost AMPK activity in cellular atrophy and stress models, improving the expression of sirtuin-1 (SIRT1), a crucial AMPK activator [184].

Recent research suggested that vit D can also trigger skeletal muscle hypertrophy and promote protein synthesis via the mTOR Complex 1 (mTORC1) signaling pathway. Overexpressing VDR in rats caused muscle hypertrophy, which was indicated by an increase in the muscle cross-sectional area, and improved anabolic signaling and translational efficacy, leading to an increase in phosphorylated mTOR and downstream targets [185]. 

Senescence is an important control measure for cell proliferation, but senescent cells create a pro-inflammatory milieu known as the senescence-associated secretory phenotype (SASP); vit D-deficient mice present cellular senescence and increased production of SASP components [186]. In addition, there is a wealth of data showing a link between low 25(OH)D levels and fat mass, and people who are overweight and deficient are more likely to experience deficits in muscle mass and function [187].

Vit D also plays an important role in mitochondrial functionality. 1,25(OH)2D3 enhanced mitochondrial bioenergetics in human skeletal muscle cell in in vitro models [188], increasing oxygen consumption rates and fission/fusion dynamics [189], while diet-induced 25(OH)D3 deficiency in animals induced impaired mitochondrial function without alterations in mitochondrial protein content [190]. Ryan et al. demonstrated that 1,25(OH)2D, but not 25(OH)D, and vitamin D3 administration increased oxygen consumption rate, highlighting the need for future studies in order to clarify effect of VDR expression and various vit D analogs and dosages on mitochondrial dynamics [189]. Vit D deficiency may cause muscle atrophy, also increasing mitochondrial ROS generation, and decreasing mitochondrial ATP synthesis [191]. A lack of vit D promotes skeletal muscle protein and lipid peroxidation [192,193], and causes impairment in the activity of antioxidant enzymes [192,194].

Mitochondria also play a crucial role in controlling SC activity; quiescent SCs contain fewer mitochondria and a lower oxidative capacity than active SCs, and the generation of ROS is known to stimulate symmetric division and terminal differentiation, supporting a role of mitochondria in affecting SC activity [195]. Vit D-induced reduced proliferation may help to maintain SC quiescence and, consequently, the stem cell population in muscle. On the other hand, the rise in metabolic machinery synthesis that comes with differentiation and the creation of mature myotubes is probably powered by an increase in mitochondrial oxygen consumption rate [196].

Vit D deficiency is indicated by a value of 20 ng/mL or less, whereas its insufficiency is indicated by a concentration of 30 ng/mL [197].

Age-related decreases in blood 25(OH)D concentration lead to decreased bone density, which increases the risk of bone fractures and falls. In research by Okuno et al., a sample of 80 elderly Japanese women over the age of 65 were found to have vit D insufficiency in 89% of cases, and deficiency in 28% [198]. A total of 56.3% of those with low or deficient vit D levels fell during the course of a three-month monitoring period. Reduced outdoor activity with age causes a fall in the body’s capacity to synthesize vit D, as well as a two-fold reduction in the skin’s generation of pre-vitamin D [199]; furthermore, the kidneys’ decreased capacity to produce 1,25(OH)2D during aging has been demonstrated [200]. Supplementing with vit D is more beneficial when serum 25(OH)D levels are low, as they often are in the elderly [201], and vit D is generally safe, since to cause hypervitaminosis D (which leads to nephrocalcinosis, hypercalcemia, and renal failure), excessive vit D ingestion over a sustained period of time is needed.

In a metanalyses on vit D supplementation, a dose of 700–1000 IU a day reduced the risk of falling among older individuals by 19%, while a lower dosage may not [202]. 

Houston et al. reported that 25(OH)D values over 50 nmol/L (20 ng/mL) are deemed sufficient to promote bone health [203]. Additionally, Ginde et al. reported that full suppression of the parathyroid hormone, which is released when vit D is required, occurs at a concentration > 40 ng/mL [204]. Multiple studies have shown that individuals with both the lowest and highest quartiles of blood 25(OH)D3 levels exhibit diminished muscular strength and an elevated risk of fractures and frailty; for this reason, a dose-dependent response in the form of a U-shaped curve has been postulated [205,206]. The adequate intake (AI) of vit D in Japan is 8.5 μg/day (340 IU) according to the Dietary Reference Intake (2020) [207]. The recommended daily intakes (AI) for vit D in the United States and Canada are 15 μg (600 IU) for individuals under 70 years old, and 20 μg (800 IU) for those aged 71 years and over [208]. The International Osteoporosis Foundation states that for older women to avoid falling and bone fractures, dietary vit D consumption of 20 to 25 μg /day (800 to 1000 IU/day) is necessary [209].

Even if integration with vit D is useful, especially in the elderly, a consensus regarding the ideal vit D amount per day is still lacking.

Future research on the creation of functional meals supplemented with vit D and foods fortified with vit D using nanoemulsion technology may greatly boost the bioavailability of vit D in the elderly, leading to improved vit D deficiency therapies.

### 4.2. Antioxidants

Malnutrition, chronic inflammation, vitamin deficiencies, and an imbalance in the muscle–gut axis are just a few of the factors that can lead to sarcopenia.

A meta-analysis revealed that patients with dysbiosis had a significant prevalence of sarcopenia, and that both sarcopenia and dysbiosis development result from oxidative stress and inflammatory conditions.

Free radicals and inflammatory mediators damage the mucosal barrier in the gastrointestinal tract and lead to bacterial invasion and dysbiosis. In conditions with a reduced quantity and diversity of gut microbiota bacteria, increased intestinal permeability leads to elevated myostatin in the muscle through the gut–muscle axis and induces sarcopenia [210,211,212].

Supplementing the diet with antioxidants can reduce oxidative stress, as evidenced in vitro [213].

Antioxidants include polyphenols, a wide family of compounds with one or more phenolic rings, and are naturally found in many food sources such as vegetables, wine, green tea, grapes, red fruits, and coffee [214].

Polyphenols are usually divided into four groups: phenolic acids, flavonoids, stilbenes, and lignans. These compounds are based on the number of phenol rings in their structure and the parts of the structure that bind the rings together [215].

Polyphenols show many potential mechanisms of action in disease prevention, including antioxidant, anti-inflammatory, immunomodulatory, and apoptotic activity.

Furthermore, these compounds alter the expression of inflammation-related genes (including NF- κB, nuclear factor erythroid 2-related factor 2 (Nrf2), Janus kinase/signal transducers and activators of transcription (Jak/STAT), and mitogen-activated protein kinase (MAPK)) by repressing the formation of downstream cytokines (e.g., IL-8, IL-1β, and TNF-α) and increasing the activities of intracellular antioxidants (such as heme oxygen-ase-1 (HO-1), superoxide dismutase (SOD), and glutathione peroxidase 1 (GPx)).

In addition, as prebiotics, they can promote healthy microbiota, the formation of short-chain fatty acids, and the reduction in intestinal permeability through the stabilization of tight junctions.

Due to antioxidant and anti-inflammatory properties, polyphenols can reduce muscle inflammation and modulate age-related transcriptional factors [212,216].

Curcumin is another molecule with antioxidant, anti-inflammatory, antimutagenic, antimicrobial, antiatherosclerotic (cardioprotective), lipid-modifying, and antitumor properties [215,217].

Curcumin’s multi-targeted actions have been demonstrated to be mediated by inhibiting several cell-signaling pathways, including NF-kB, STAT3, Nrf2, ROS, and COX-2 [169].

Curcumin is found in the rhizomatous herbaceous perennial plant (*Curcuma longa*) of the *Zingiberaceae* family, and curcumin is the main natural polyphenol found in the rhizome of Curcuma longa (turmeric) and other *Curcuma* spp. [218,219]. 

The acceptable daily intake for curcumin, as decided by the Joint Food and Agriculture Organization (FAO)/World Health Organization (WHO) Expert Committee on Food Additives (JECFA) and the European Food Safety Authority (EFSA), is 0–3 mg/kg body weight [220].

Despite the fact that curcumin exhibits various biological functions, its clinical application is limited due to its poor absorbability after oral administration. For this reason, innovative pharmaceutical formulations have been developed for oral administration.

After some preliminary studies using various delivery systems, the application of nanotechnology for curcumin preparation has greatly improved its oral bioavailability. Nanoparticles have enhanced the absorption of curcumin 30-fold in both rats and humans.

BALB/c mice (six- to eight-week-old females) were treated with dextran sulfate sodium (DSS)-induced colitis administration, DSS plus nanoparticle curcumin, or nanoparticle curcumin. The disease activity index was markedly higher in the DSS group than in the DSS plus nanoparticle curcumin group [169].

It has been reported that curcumin is a potent inhibitor of NF-kB activation, which induces the expression of several inflammatory genes [221].

The study results of [169] showed that the translocation of NF-kBp65 into the nucleus was significantly more suppressed in the DSS plus nanoparticle curcumin group than in the DSS group. Thus, the inhibition of NF-kB leads to the suppression of mucosal inflammation [169].

Furthermore, it has been discovered that treatment with nanoparticle curcumin elevated the population of high butyrate-producing bacteria, *Clostridium* cluster IV and XIVa, which led to increased fecal butyrate levels. Butyric acid is crucial due to its beneficial influence on intestinal homeostasis and its anti-inflammatory properties, which can increase mucosal immunity and intestinal barrier integrity [222].

In a separate research study, Peterson et al. recruited 32 adults aged 19 to 58, who were randomized into three groups: the placebo, turmeric, and curcumin tablet groups [170]. The subjects had to orally take three 6000 mg tablets daily with food, twice a day.

The authors compared the abundance of bacterial species in each group pre- and post-treatment. They observed an overall reduction in species by 15% in the placebo group, whereas subjects given turmeric showed a modest 7% (156 vs. 167) increase in the number of species seen. Notably, those receiving curcumin showed an average rise in identified species of 69%.

On the other hand, an ANOVA comparison of diversity indices did not reveal statistically significant differences between the turmeric and curcumin tablet groups because of the elevated variation within participants.

Human studies are required to further understand the effect of turmeric and its constituents on the microbiota.

In addition, Gorza et al. [171] suggested a possible muscle-specific response to curcumin treatment. In particular, chronic systemic curcumin administration significantly reduced spontaneous mortality during senescence, effectively combated presarcopenia, and significantly attenuated sarcopenia, improving satellite cell commitment and recruitment. This suggests that chronic systemic curcumin administration is very effective in aging mice.

Curcumin treatment prevented muscle mass loss, mitigated age-related soleus force loss, and reduced the mortality of old mice. Since the benefits on lifespan were also evident in aged 6J mice, who did not yet show any apparent muscle involvement, it seemed to occur independently of that in skeletal muscle. Curcumin therapy preserved the size of type-1 myofibers and promoted hypertrophy in type-2A myofibers in aged 10ScSn mice, preventing the onset of soleus sarcopenia [171]. Interestingly, flavonoid quercetin is also an excellent antioxidant, and its therapeutical properties include anticancer and anti-inflammatory effects, and the prevention of cardiovascular disease [223,224]. Furthermore, recent studies show that quercetin significantly affects diabetes and obesity [225].

In a study by Zhang et al. quercetin revealed anti-inflammatory and immunomodulatory properties in vitro. So, Cacumen Platycladi, which contains quercetin, was selected for the in vivo experiment. Mice who received quercetin and Cacumen Platycladi demonstrated improved intestinal structure, less diarrhea, and a lower incidence of fecal occult blood. In general, pyroptosis and inflammation in mice’s intestines were significantly reduced by the aqueous extract of Cacumen Platycladi and quercetin [226].

An experimental study by Ju et al. 2018 demonstrated that quercetin intake with diet could partially alleviate colitis by modulating the anti-inflammatory effects and bactericidal capacity of macrophages via the HO-1-dependent pathway. This suggests that quercetin might restore the proper intestinal host–microbe relationship to ease colitis by rebalancing enteric macrophages’ pro-inflammatory, anti-inflammatory, and bactericidal functions. Hence, restoring immunological homeostasis and rebalancing the enteric commensal flora through the dietary administration of quercetin to modulate the function of intestinal macrophages is a potential and promising approach for treating inflammatory bowel disease [227].

On the other hand, an experimental investigation by Kanzaki et al. [28] used quercetin as a sarcopenia therapy. Male C57BL/6J (B6) mice were given quercetin glycosides (QG) in drinking water at two dosages (1.5 or 3.0 g/L) for 24 weeks. Grip strength and rotarod performance were significantly improved in treated mice. Increased wet weights of a particular group of muscles (quadratus femoris, gastrocnemius, tibialis anterior, and soleus) in the treated mice’s muscular morphology demonstrated functional benefits. Hence, the long-term oral administration of QG during the early stages of aging can safely and effectively enhance certain aspects of motor performance and increase muscle mass [172].

Furthermore, another compound considered to have strong antioxidant, anti-inflammatory, and metabolic regulatory properties is resveratrol [228].

Resveratrol can significantly modify the composition of the gut microbiota by preventing the growth of specific microbial species or creating a shift in the bacterial population [85].

In particular, resveratrol has been shown to have anti-inflammatory properties in intestinal cells. One study examined the protective effects of resveratrol in different concentrations (10–50 µg/mL) of Caco-2 cells exposed to lipopolysaccharides generated from bacteria. It was demonstrated that decreased COX-2 expression was associated with lower PGE2 levels in cells treated with resveratrol. Moreover, resveratrol prevented NF-kB activation by slowing the rate at which IkB, an endogenous NF-kB inhibitor, degrades [229].

Another study demonstrated that resveratrol at high concentrations (440 µm) protected Caco-2 cells against indomethacin-induced mitochondrial dysfunction [230,231].

In addition, in some experimental studies, resveratrol has been shown to increase muscle protein synthesis, decrease muscle protein degeneration, and attenuate skeletal muscle fiber atrophy. For example, a significant decrease in muscle fiber atrophy was recorded in rodents given 400 mg/kg of resveratrol daily [173]. A low dose of resveratrol (12.5 mg/kg/day) did not increase the number of satellite cells or muscle mass. However, other data on the ingestion of resveratrol with exercise showed that combining this natural compound with training reduced sarcopenia in humans to a greater extent than exercise alone [217].

### 4.3. Omega-3 Fatty Acids 

Ω-3 fatty acids (FAs) are polyunsaturated Fas (PUFAs); they include docosahexaenoic acid (DHA), eicosapentaenoic acid, and docosapentaenoic acid, and are mainly contained in fish meat, eggs, seafood, and vegetable oil [85]. Ω-3 fatty acids have been linked to improvements in the composition and diversity of the gut microbiome in middle-aged and older women, and the concurrent administration of ω-3 fatty acids and probiotic strains provides amplified health benefits [232].

Ω-3 polyunsaturated fatty acids have been associated with attenuating inflammation in IBD, probably acting as substrates for producing anti-inflammatory eicosanoids. These compounds have similar structures and properties to prostaglandins and leukotrienes [233].

These fatty acids influence disease development by reducing oxidative stress, producing TNF-α and proinflammatory cytokines, working as chemoprotective agents, and reducing the expression of adhesion molecules in intestinal cells.

Diets containing high levels of ω-3 PUFAs improved inflammation and mucosal damage in experimental rat models of UC, and specific fatty acids were predicted to be used as therapeutic drugs [234].

New evidence is available about the efficacy of ω -3 fatty acid dietary supplementation to improve protein metabolism and counteract anabolic resistance through indirect effects. Studies show that long-term fish oil administration can enhance the anabolic stimuli from substrates (e.g., aminos and/or proteins), hormones (e.g., insulin), and/or physical activity in skeletal muscle [235].

Furthermore, some studies have found that linolenic acid, a common n-3 polyunsaturated fatty acid (n-3 PUFA), might improve sarcopenia. In this study, *Caenorhabditis elegans (C. elegans*) was used as a model animal to investigate the effects of linolenic acid on *C. elegans* muscles. The results showed that 50 μg mL-1 linolenic acid significantly improved sarcopenia by repairing mitochondrial function by promoting mitophagy and fighting oxidative stress [174].

### 4.4. Probiotics

An increasing number of studies provide evidence that muscle function can be modulated by the gut microbiota [148]. Several animal model studies provide evidence of the effects of probiotics on the onset and progression of age-related muscle impairment via the gut–muscle axis.

Chan and colleagues provide evidence of the effects of probiotics on the onset and progression of age-related muscle impairment via the gut–muscle axis [236].

Their research was conducted on senescence-accelerated mouse prone-8 (SAMP8), who were administered *Lactobacillus casei* Shirota (LcS) (1 × 10^8^ or 1 × 10^9^ CFU/mouse/day) for 12 weeks. Afterwards, muscle mass and oxygen consumption rate were measured, and maintenance pulse and grip force tests for muscle strength were performed.

Chen demonstrated that the administration of LcS probiotics in aged SAMP8 mice can regulate microbiota composition and, in particular, it was observed that bacterial strains related to younger muscle conditions were regulated. In addition, LcS administration reduced the levels of signaling mediators involved in the age-related gut–muscle axis, such as inflammatory cytokines, and in SCFAs [236]. Finally, the maintenance of mitochondrial and muscle functions is noteworthy.

Given the correlation between intestinal microbiota and muscle function, these findings suggest that LcS could regulate the onset of age-related sarcopenia through the gut–muscle axis [237]. Changes to intestinal microbiota brought about by LcS cause changes in the production of mediators such as SCFA [149], cytokines and ROS, which maintain mitochondrial function, and thus, delay the progression of muscle aging.

In addition, Lee et al. investigated the supplementation of *B. longum* OLP-01 in mice from whom it had been eliminated from the gut microbiota by antibiotics and who had an impaired ability to contract their external skeletal muscles. The consecutive administration of this strain successfully affected grip strength and increased resistance in mice in a dose-dependent manner [150].

The supplementation of 12 weeks of *L.paracasei* PS23 was also performed in SAMP8 mice divided into three groups (n = 6 each): non-aging (16 weeks old), control (28 weeks old), and PS23 (28 weeks old). The main results obtained evidenced that LPPS23 extenuated sarcopenia progression during aging; this effect might have been enacted by preserving the mitochondrial function via a reduction in age-related inflammation and ROS, and by retaining protein uptake in the SAMP8 mouse model [154]. 

An increased rate of oxygen consumption in the muscle and mitochondrial biogenesis have been also observed.

Supplementation of several LAB strains (*Limosilactobacillus fermentum* DR9, *L. paracasei* OFS 0291, or *L. helveticus* OFS 1515) over 12 weeks was performed in an early aging model of Sprague Dawley rats; this treatment led to an increase in exercise performance following an exhaustion test on a treadmill compared to untreated rats [238]. Likewise, the authors highlighted a decrease in senescence markers, such as the tumor suppressor protein p53, and an increase in IGF-anabolic factors comparable with young rats in the muscles of supplemented elderly rats.

Synbiotics, a combination of prebiotics (non-digestible fiber) and probiotics, also provide an emerging nutritional strategy to promote the development of specific species of gut microbiota. In addition, synbiotics can suppress low-grade inflammation through the administration of SCFA, mediated by an increased community composition of colonial bacteria in older adults [151,152].

Although studies on animal models are numerous, few human studies have been published to date.

Lee and colleagues, in 2021, reported that the administration of probiotic *L. plantarum* TWK10 in mice could prevent muscle weakness associated with aging, bone loss, and cognitive deterioration by improving the concentration of glycogen in muscle tissue and by modulating the intestinal microbiome [153].

Previously, the same supplementation in mice for six weeks reported an improvement in muscle mass and exercise performance through a reduction in inflammation and markers of muscle atrophy [239].

Consistent with this, a randomized double-blind clinical study conducted by Lee and colleagues showed that six-week oral administration of *L. plantarum* TWK10 could lead to improved muscle mass and function in fragile older adults, thus preventing sarcopenia [240].

Several experimental studies have shown that microbial enrichment accompanied improved insulin sensitivity [241], grip strength, and fragility conditions [242]. Furthermore, increased SCFA concentrations [243] and a reduction in proinflammatory cytokines [244] have been also observed in both young and elderly adults.

Finally, kefir is also a valuable probiotic. Specifically, kefir is an acid-fermented milk with traces of alcohol containing lactic acid bacteria and yeast. Research into the beneficial effects of Kefir has increased over the last decade [245], making kefir an important functional dairy product with multiple biological activities [246].

The beneficial properties of kefir against fatigue were investigated by Hsu et al. by analyzing modulation of the composition of the gut microbiota [155]. 

Four groups of eight male Institute of Cancer Research (ICR) mice each received different daily doses of kefir for 4 weeks. By measuring exhaustive swimming time and forelimb grip strength, the impacts of the various doses of kefir on fatigue and physical exercise performance were evaluated, with the authors observing significant improvement in the additional groups compared to the control groups. In the same way, the composition of the gut microbiota was also altered, with the authors finally finding a correlation between supplementation modification of the gut microbiota and exercise [155].

Although studies have been carried out on animal models, in light of these results, it can be assumed that improvement in the health of the gut microbiota related to kefir supplementation could be a valid support strategy to improve age-related muscle frailty syndromes such as sarcopenia.

### 4.5. Prebiotics

Few studies have examined the beneficial effects of prebiotics on the health of older people. Most prebiotics used in studies of older people are natural polysaccharides, fructooligiosaccarides (FOS), inulin, and galactooligosaccharides (GOS). The main gut microflora targets of these prebiotics are *Bifidobacterium* spp. and *Lactobacillus* spp. [156,247].

Previous studies have shown that species beneficial for health were increased, and at the same time, deleterious species were decreased, in the microbiota as a result of FOS and inulin intake [156,157].

In addition, fitness, nutritional status, quality of life, and frailty were also improved after 12–13 weeks of nutritional supplementation with FOS and inulin [242,248].

Supplementation for 10 weeks with GOS may also alter the gut microbiota and immune response [249], and the consumption of GOS for 3 weeks increases *Bifidobacteria* spp and the production of butyrate; this highlights all the benefits it can bring this to muscles [158,159].

Tomiaga and colleagues, in their study, analyzed [250] the effects of 1-kestose on changes in the intestinal microbiota, observing how the population of *B. longum* increased significantly in the intestines of six subjects after 12 weeks of taking 1-kestose. In addition, this supplement increased the index of skeletal muscle mass while reducing that of body fat. The administration of a prebiotic led to the recovery of muscle atrophy in super-elderly patients with sarcopenia.

In addition, in a placebo-controlled, randomized, double-blind project conducted by Buigues et al. [248] on sixty elderly subjects aged 65 years and over, the authors observed beneficial responses of a prebiotic compound of a mixture of inulin plus fructooligosaccharides (maltodextrin). Participants were randomized for parallel group surgery lasting 13 weeks with a daily dose of prebiotic or placebo.

The results showed a beneficial effect on the muscle strength of the hand after the administration of prebiotic mix compared to the placebo group, suggesting that prebiotics can affect the muscle system. Prebiotics could therefore be included in the total treatment protocol of people suffering from age-related conditions, especially frailty, and as a preventive intervention in general. 

### 4.6. Protein

Several clinical studies investigating the potential therapeutic effects of nutrition against sarcopenia have been inspired by the encouraging results of protein nutritional support in cellular and animal models studies.

For aged people or patients with wasting disorders such as cancer, protein dietary supplementation plus resistance training is typically given in the clinic to increase muscle mass. In comparison to the control group, protein supplementation boosted myofibrillar protein synthesis in a study of healthy women (aged 65 to 75). Those who consumed 15 g of whey protein containing 4.2 g of leucine showed significantly improved acute myofibrillar protein production [251].

Another study showed that whey protein supplementation enhanced lower limb strength and muscular function in elderly patients being treated in a geriatric unit. Although the lower limbs were the obvious focus of this investigation, it is possible that the effect could extend to the subject’s entire voluntary musculoskeletal system [252].

Clinically cancer-free people frequently experience a loss of body mass due to significant androgen hormone deprivation, especially those with prostate cancer.

Resistance training plus protein supplementation (50 g of whey protein isolate) significantly prevented the loss of lean mass in the treated subjects.

Autophagy, proteolysis, and myostatin-mediated anabolic resistance were found to be elevated in a clinical trial involving patients with alcoholic cirrhosis. The administration of a leucine-enriched branched-chain amino acid mixture substantially decreased the expression of the autophagy-related protein mTOR, and subsequently reversed autophagy-induced muscle proteolysis, in comparison to individuals in the control group. These findings showed that oral nutritional supplements significantly reduced the rate of illness and weight loss. Moreover, a recent study found that circulating miR-203 contributed to the promotion of myopenia in people with colorectal cancer [253].

After whey protein intake, a subject with leg immobilization also showed altered miRNA levels. *MiR*-208b expression was inhibited and *miR*-23a expression was increased, which can prevent sarcopenia brought on by immobility.

Further clinical trials showed that total protein leucine content played a crucial role in increasing skeletal muscle’s anabolic response. In two groups of elderly people, the first group received a supplement of 25 g of whey protein isolate containing 3 g of leucine, while the second group received 10 g of whey protein isolate containing 3 g of leucine. Comparable results on maintaining skeletal muscle protein synthesis and improving muscle loss were obtained. From this study, it was understood that a higher concentration of leucine, for the same amount of total protein, was able to promote more myofibrillar protein synthesis [254].

Patients receiving blended soy and whey proteins experienced improved grip and arm muscular strength. Generally, aged people and patients may benefit from nutritional support that is high in protein, particularly a diet abundant in leucine, because it can effectively slow down muscle loss and increase muscle function. Due to its appetite-suppressing effects and anabolic effects that sustain muscle protein synthesis (MPS) over muscle degradation (MPB), protein is the predominant macronutrient in weight reduction techniques in obese and sarcopenic people [255].

The secretion of peptide YY (PYY) and glucagon-like peptide-1 (GLP-1) is modulated by G receptors coupled to proteins (GPCRs), which are found in L and G cells of the colon and small intestine, respectively [256,257]. Cholecystokinin (CCK), which is released in response to protein consumption, also contributes to the feeling of fullness. Regarding the consumption of carbohydrates and fats in the diet, which is thought to be related to the regulation of leptin and ghrelin secretion, several studies have supported these effects. However, appetite-induced responses, which are influenced by signals between the dietary proteins of the intestinal microbiota, can be determined by the composition of amino acids and, in particular, by essential amino acids, such as leucine [258]. Low-protein diets reduce the satiating and anabolic benefits of dietary proteins in elderly people [259]. Because older people have a lower ability than younger people to absorb and utilize protein, the current Recommended Dietary Allowance (RDA) for proteins, which is 0.8 g/kg/day, may not be enough for them [160].

The recommended ingestion of leucine is approximately 3–4 g per meal, which is equivalent to 25–30 g of high-quality protein and 1.0–1.6 g/kg/day spread over 3–4 meals per day [260]. This is aimed at promoting further stimulation of MPS in the elderly.

Intestinal microbial diversity is influenced by dietary protein sources and their amino acid composition. To be more specific, we know that eating plant proteins is linked to higher levels of *Bifidobacterium*, *Roseburia*, *Ruminococcus bromii*, *Lactobacillus*, and *Roseburia* [106], whereas eating animal proteins is linked to higher levels of *Bacteroides*, *Alistipes*, *Bilophila*, and *Clostridium perfrigens* [261].

It is noteworthy that rich soy protein, mung bean, and buckwheat diets have also been associated with increased bile acid transformation and GLP-1 secretion, high levels of *Lactobacillus* and *Bifidobacterium*, and lower *Firmicutes* [262]. Moreover, *Bacteroides fragilis* and *Clostridium perfingens* populations have been shown to decrease because of whey and cheese proteins fermented with *Bifidobacteria*, whereas acetate synthesis and the diversity of *Lactobacillus* and *Bifidobacterium* have been shown to increase [263].

Moreover, several species of *Lactobacillus* and *Bifidobacteria* have been linked to improved muscle mass, decreased body weight, and decreased obesogenic settings in both people and mice [264]. This is explained by the high levels of whey protein seen in *Lactobacillus* and *Bifidobacteria* in rodent studies [265]. Regarding this, the ingestion of white meat proteins was shown to enhance the number of *Lactobacillus*, and supplementing mice’s diets with *L. plantarum* led to an increase in their muscle mass [265].

Increased SCFA content and decreased *Proteobacteria* (*Helicobacter*) were observed in mice fed with fish protein [266].

When administered to endurance athletes for 70 days, a whey protein supplement containing beef (25 g) decreased *Roseburia*, *B. longum*, and *Blautia*, while increasing the species of *Bacteroidetes* [267]. This was in contrast to the control group receiving maltodextrin.

However, in another study, the integration of high-protein beef in germ-free mice (GF), compared to mice with an unchanged microbiome, showed an improvement in grip strength in both groups [132], leading the authors to question the effects of feeding high-protein beef to mice with different microbial compositions.

Interestingly, a more favorable microbial composition has been associated with increased consumption of plant proteins, compared to animal proteins, partly due to the higher percentage of SCFA-producing bacteria.

### 4.7. SCFAs

Increasing evidence suggests a role of SCFAs in regulating muscular functions and mass through modification of the microbiota [268].

In 2022, a randomized, double-blind, controlled study investigated the effects of 12-week supplementation with oral β-hydroxy-β-methyl butyrate (HMB) on changes in the body composition of 43 subjects.

The results showed an upward trend in grip strength in the HMB group. This could indicate that β-hydroxy-β-methyl butyrate (HMB) supplementation increases muscle mass and strength in some muscle wasting disorders.

Other studies were conducted on humans to support these data, collected in a meta-analysis study by Holeček [161].

Based on a meta-analysis of seven randomized controlled trials [162,163,164,165,166,167,168], it has been shown that supplementation with HMB in the elderly can prevent the loss of lean body mass without causing a significant change in fat mass. These results suggest that supplementation with HMB could mitigate the marked decreases in skeletal muscle mass and function that occur with aging.

## 5. A Non-Pharmacological Integrated Approach against Sarcopenia

**Figure 1:**
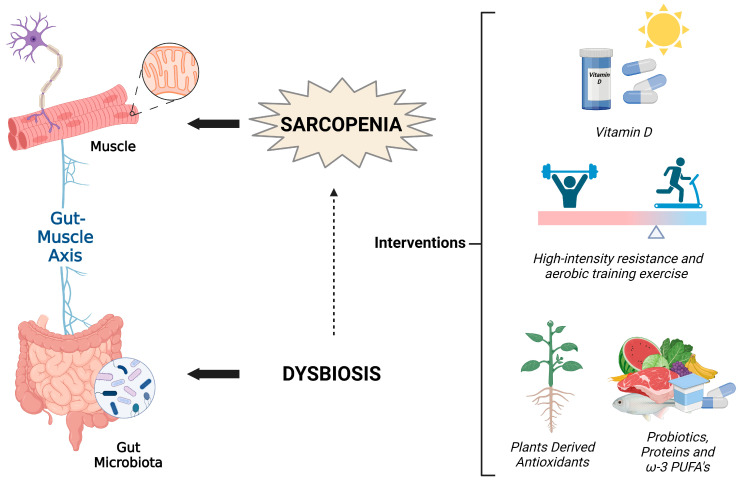
A comprehensive approach to an integrated intervention to preserve musculoskeletal health in aging.

Sarcopenia, the age-related decrease in muscle mass and function, has a significantly negative impact on people’s lives and on society as a whole, and is a common condition among elderly patients admitted to hospitals [269]. An integrated strategy that considers the complex etiology of sarcopenia and aims to prevent and counteract this condition in the elderly is essential. In this review, we focused on the influence of lifestyle and specific supplements on the maintenance of skeletal muscle health in the elderly, considering their specific features. The described approaches can act synergistically in order to prevent and ameliorate sarcopenia (Figure 1).

With advancing years, elderly people typically experience a reduction in their nutritional and energy consumption [270,271] due to the “anorexia of aging” [272]; furthermore, inflammation, whether acute or chronic, can raise energy needs, which can result in “disease-related malnutrition” [273]. Indeed, the diets of the elderly are low in protein, and supplementation with amino acids and protein can represent an important strategy in the prevention of sarcopenia due to their effects on skeletal muscle and bone health [274]. Protein in the diet plays a fundamental role in the modulation of the microbiota [132]. A study on rats has shown that feeding with vegetable (soy) or animal (derived from fish) protein favors the condition of eubiosis. At the same time, a diet composed of foods high in fats adversely affects the intestinal microbiota, as has been shown in many studies [90]. Protein-based nutritional support leads to an increase in the abundance of *Bifidobacteria* and *Lactobacilli*, helping to maintain intestinal health and prevent overweight and obesity. Nutritional support effectively restores the composition of the intestinal microbiota [275]. In rats fed a low-calorie diet, a decreased abundance of *Firmicutes* and an increase of the levels of *Bacteroidetes* and *Proteobacteria* have been observed [276]. Protein nutritional support, such as a whey protein diet, is beneficial for maintaining the gut microbiota and intestinal health as it modulates the abundance of *Firmicutes, Bacteroidetes*, and *Proteobacteria* [277]. A strategy that aims to maintain a healthy gut microbiota represents an important aid in healthy aging (Figure 1) [85].

Since vit D deficiency might result in gastrointestinal disease due to its immunomodulatory properties, a connection between vitamin D and gut microbiota has recently been proposed. Recent studies on both humans and animals have revealed that vit D could modulate the composition of the microbiota, preserving gut homeostasis [278] and reducing gut permeability [279]. Vit D alters the microbiota, causing *Firmicutes* to decline and *Bacteroidetes* to increase, and increasing beneficial bacteria such as *Ruminococcaceae, Akkermansia, Faecalibacterium*, and *Coprococcus* [280].

Another essential element of sarcopenia management and prevention is exercise (Figure 1). Exercise has been shown to modulate microbiota as well, improving beneficial species and increasing their diversity. The gut microbiota, which controls energy balance and contributes to the control of inflammatory, redox, and hydration status, may, on the other hand, have an impact on adaptation to exercise [281]. The interaction between vit D and physical activity in sarcopenia was investigated by Yang et al. [282]. The authors reported that in the elderly, the effect of vit D on muscle strength depends on physical activity level, suggesting a synergistic effect. Detailed knowledge of the effects of different nutrition, supplementation, exercise, and microbiota-based strategies, and of their synergic action on sarcopenia, will allow for the development of a combined strategy.

## 6. Conclusions

A sedentary lifestyle and poor diet are important risk factors for sarcopenia, which afflicts many elderly people. An integrated approach involving training programs designed to improve muscle mass, adequate nutrition that aims to modulate the gut microbiota and general health, and eventual supplementation with vit D antioxidants, PUFAs, vitamins, probiotics, prebiotics, proteins, kefir, and short-chain fatty acids could be useful in counteracting sarcopenia. It is important to highlight that a personalized strategy is essential, especially when targeting a particular and varied population such as the elderly who are at risk of sarcopenia or sarcopenic conditions. Older people need a well-designed and carefully administered training program and a diet with or without supplementation calibrated to their specific needs. 

Today, there is great interest in personalized medicine tailored to the unique characteristics of individuals. The same degree of attention to individual features, made possible by technological progress, should also be paid to defining non-pharmacological intervention, to avoid side effects and maximize benefits. Deep characterization of the subject that takes into account general condition, motility problems, nutritional habits, vit D deficiency, and the microbiota population is needed in order to detect the best integrated strategy. Furthermore, the evolution of the situation should be monitored in order to ensure the best follow-up treatment.

## Figures and Tables

**Table 1:** Supplements demonstrating efficacy through specific doses able to maintain musculoskeletal health.

Supplements	Model	Sample Size	Administration (Dose/Day and Duration) of Supplementation	General Effects and Possible Mechanism of Action	Effects on Microbiome Composition	Refs.
**Probiotic**						
*Lactobacillus casei* Shirota	SAMP8 mice	24	1 × 10^8^ or 1 × 10^9^ CFU/mouse/day × 12 weeks	↓ signaling mediators (inflammatory cytokines and SCFAs); = mitochondrial and muscle functions	↑ *Odoribacter*, *↑ Oscillibacter*, *↑ Lachnospiraceae UCG 006*	[148]
*Bifidobacterium longum* OLP-01	ICR mice	40	2.05 × 10^9^; 4.10 × 10^9^; and 1.03 × 10^10^ CFU/kg/day for 4 weeks	↓ lactate, ammonia, and CK levels	↑ *Lactobacillus*, ↑ *Clostridium*, ↑ *Bifidobacterium*, ↑ *Lactococcus*, ↓ *Bacillus*, ↓ *Enterococcus*, ↓ *Akkermansia*	[149]
*Lacticaseibacillus paracasei* PS23	SAMP8 mice	80	1 × 10^9^ CFU/mouse/day for 12 weeks	↑ lean mass, restored muscle strength, ↑ SOD, ↑ gpx in muscles		[150]
*Lactobacillus plantarum* TWK10	ICR mice	33	1 × 10^9^ CFU/mouse/day for 6 weeks	Prevention of muscle weakness associated with aging, ↑ concentration of glycogen in muscle tissue	*↓ Enterobacteriaceae*, *↓ Enterococcaceae*, *↑ Lactobacillaceae*	[151,152]
*Lactobacillus plantarum* TWK10	ICR mice	24	2.05 × 10^8^, 1.03 × 10^9^ units/kg/day for 6 weeks	↑ muscle mass (gastrocnemius muscle), ↑ energy harvesting, health promotion, performance improvement, anti-fatigue effects		[153]
*Limosilactobacillus fermentum* DR9	Sprague Dawley rats,	18	10 log CFU/rat/day for 12 weeks	↓ p53, increase in IGF-1 anabolic factors, prevention of aging-induced metabolic diseases	Inhibited growth of pathogens *Escherichia coli*, *Staphylococcus aureus*, and *Staphylococcus epidermidis*	[154]
*Lactobacillus Paracasei* OFS 0291						
*Lactobacillus helveticus* OFS 1515						
**Kefir**	ICR mice	32	2.15, 4.31, and 10.76 g/kg/day for 4 weeks	↑ exercise performance, ↑ forelimb grip strength, ↑ swimming time to exhaustion	↓ *Firmicutes/* *Bacteroidetes* ratio	[155]
**Prebiotics**						
FOS and inulin	Human	50	one spoon/ day for 13 weeks	↑ levels of fatigue, ↑ muscle strength		[156,157]
GOS	Human	39	4 g/ twice daily for 3 weeks	Production of butyrate	↑ *bifidobacteria*, ↑ *lactobacilli*	[158,159]
1-kestose	Human super-elderly patients	6	10 g/day for 12 weeks	↑ index of skeletal muscle mass, ↓ body fat	↑ *Bifidobacterium longum*	[158,159]
**SCFAs**						
β-hydroxy-β-methylbutyrate (HMB)	Human	43	1.5 g twice daily for 12 weeks	↑ muscle mass, ↑ muscle strength		[160]
HMB/Arg/Lys mixture	Human	77	2 g/5 g/1.5 g per day) for 1 year	↑ lean tissue mass, ↑ protein turnover		[161]
HMB	Human	79	2 g/day for 2 or 4 weeks	↓ urinary urea nitrogen excretion		[162,163,164,165,166,167,168]
HMB	Human	31		↑ body fat loss		[162,163,164,165,166,167,168]
HMB/Arg/Lys mixture	Human	57	2/5/1.5 g per day for 12 weeks	↑ limb circumference, ↑ leg and handgrip strength, ↑ body protein synthesis		[162,163,164,165,166,167,168]
HMB/Arg/Lys mixture	Human	77	2/5/1.5 g per day for 1 year	↑ muscle mass, ↑ muscle strength		[162,163,164,165,166,167,168]
HMB/Arg/Gln mixture	Human	35	4 g HMB/14 g Arg/14 g Gln for 2 weeks	↑ collagen synthesis in muscle		[162,163,164,165,166,167,168]
HMB	Human	80	(1.5 g/day) + mild fitness program for 8 weeks	↑ muscle strength, ↑ physical performance parameters		[162,163,164,165,166,167,168]
**Curcumin**						
	BALB/c mice		0.2% (*w*/*w*) nanoparticle curcumin mixed with normal rodent diet for 7 days	Suppression of the activation of NF-kB	↑ butyrate-producing bacteria and *Clostridium* cluster IV	[169]
	Human	32	6 tablets daily; 6000 mg for 4/8 weeks		↑ *Clostridium*, ↑ *Bacteroides*, ↑ *Citrobacter*, ↑ *Cronobacter*, ↑ *Enterobacter*, ↑ *Enterococcus*, ↑ *Klebsiella*, ↑ *Parabacteroides*, ↑ *Pseudomonas*, ↓ *Blautia*, ↓ *Ruminococcus*	[170]
Curcumin + HPβCD	C57BL6J and C57BL10ScSn mice	143	every sixth day, for 6 months, with120 μg/kg	↑ satellite cell commitment and recruitment		[171]
**Quercetin**						
Quercetin glycosides	Male C57BL/6J mice		Two dosages (1.5 or 3.0 g/L) for 24 weeks	↑ Strength and rotarod performance, ↑ wet weights		[172]
**Resveratrol**	Wistar rats		400 mg/kg/day	↓ muscle fiber atrophy		[173]
**Ω-3 fatty acids**						
Linolenic acid	Caenorhabditis elegans		50 μg mL^−1^	repair of mitochondrial function by promoting mitophagy and fighting oxidative stress		[174]

CFU = colony-forming units; CK = creatine kinase; SOD = superoxide dismutase; IGF-1 = insulin-like growth factor-1; ***↑*** = increase; **↓** = decrease.

## Data Availability

Data sharing not applicable.
